# Isolation of Marine-Derived Microorganisms for PET Biodegradation

**DOI:** 10.3390/microorganisms14081804

**Published:** 2026-08-16

**Authors:** Shijing Deng, Qiaoqiao Guo, Yunhe An, Yuqing Liu, Jianping Yin, Songbiao Shi, Tingbiao Wu, Chenlu Gu, Xinpeng Tian, Qinglian Li

**Affiliations:** 1 State Key Laboratory of Tropical Oceanography, Guangdong Key Laboratory of Marine Materia Medica, South China Sea Institute of Oceanology, Chinese Academy of Sciences, Guangzhou 510301, China; dengshijing23@mails.ucas.ac.cn (S.D.); guoqiaoqiao@scsio.ac.cn (Q.G.); anyunhe24@mails.ucas.ac.cn (Y.A.); liuyuqing232@mails.ucas.ac.cn (Y.L.); yjp@scsio.ac.cn (J.Y.); sbshi@scsio.ac.cn (S.S.); wutingbiao@szbl.ac.cn (T.W.); cheryl_chenlu@foxmail.com (C.G.); 2 Department of Marine Pharmacy, School of Life Science and Biopharmaceutics, Guangdong Pharmaceutical University, Guangzhou 510006, China; 3 University of Chinese Academy of Sciences, Beijing 100049, China

**Keywords:** polyethylene terephthalate, marine environments, plastic biodegradation

## Abstract

The long-term accumulation of polyethylene terephthalate (PET) in marine environments may drive the evolution of microbial degradation capabilities, positioning the ocean as a valuable reservoir for discovering novel PET-degrading microorganisms. In this study, we isolated 305 marine-derived microorganisms with potential PET-degrading capability from samples collected from mangrove areas of Zhanjiang and the intertidal zones of Daya Bay, Shenzhen, China, using PET powder as a major carbon source. Subsequent evaluation of degradation performance via scanning electron microscopy and Fourier-transform infrared spectroscopy analysis identified 14 isolates capable of degrading PET film. These 14 strains belonged to 14 distinct species, none of which, to the best of our knowledge, has been previously documented as PET degraders. Among them, *Microbacterium aurum* SCSIO 85700 exhibited the most potent PET-degrading activity, achieving a weight loss of 2.1 mg (2.1%) and a 6.5% increase in relative crystallinity over 30 days. Genome analysis revealed the genetic basis underlying PET degradation and associated metabolic pathways in strain SCSIO 85700. Notably, genome mining and structural modeling identified two candidate polyester hydrolases, MA2267 and MA2443, possessing conserved His–Asp–Ser catalytic triads and exposed substrate-binding clefts resembling those of characterized PET-degrading enzymes, suggesting their potential involvement in PET depolymerization. Collectively, this study expands the recognized diversity of marine PET-degrading microorganisms and provides microbial resources for sustainable PET bioremediation.

## 1. Introduction

Since its first synthesis in the early 20th century, plastic has become an indispensable synthetic material supporting modern industrial development and daily life [1]. Among various plastic polymers, polyethylene terephthalate (PET) is one of the most widely produced plastics [2]. PET is synthesized through the polycondensation of terephthalic acid (TPA) and ethylene glycol (EG), resulting in characteristics such as high mechanical strength, excellent chemical resistance, low gas permeability, and outstanding thermal stability [3]. Due to these favorable properties, PET is extensively used in textiles, household goods, and packaging [4]. Global PET production currently exceeds 70 million tons annually and continues to increase in response to growing consumer demand [5]. However, the intrinsic recalcitrance of PET, arising from its highly stable aromatic polyester backbone and semi-crystalline structure, makes it resistant to natural degradation processes [4]. Consequently, enormous quantities of post-consumer PET waste accumulate in terrestrial, freshwater, and marine environments [6]. Fragmentation of discarded PET materials further generates microplastics that can persist for decades, posing significant ecological and environmental concerns [7]. Therefore, the development of efficient and sustainable PET waste management strategies has become a critical global challenge [8].

To address PET pollution, various recycling and degradation technologies have been developed, including mechanical recycling, chemical depolymerization, thermal treatment, and biological degradation [4,9]. Among these methods, bio-based degradation using microorganisms or their enzymes offers a green and sustainable route [9]. Research on PET biodegradation has advanced over the years. A milestone discovery was reported by Yoshida et al., who isolated a bacterium, *Ideonella sakaiensis* 201-F6, and demonstrated its ability to utilize PET as a major carbon and energy source [10]. This strain secretes a PET hydrolase, *Is*PETase, capable of hydrolyzing PET into the monomer mono(2-hydroxyethyl) terephthalate (MHET) and TPA, representing the first naturally occurring PET hydrolase shown to efficiently depolymerize PET under ambient conditions. Another important breakthrough was the discovery of leaf-branch compost cutinase (LCC), a highly thermostable PET-degrading enzyme identified from a leaf-branch compost metagenome [11]. The discovery of *Is*PETase and LCC stimulated extensive protein-engineering efforts, yielding a number of engineered PET hydrolases with improved activity and enhanced thermostability [12,13]. Despite these advances, the vast genetic diversity and abundance of microorganisms in nature suggest that many more PET-degrading species remain undiscovered.

Marine microorganisms represent a particularly promising reservoir for discovering novel PET-degrading bioresources. It is estimated that around 85% of PET waste bypasses collection systems and resides in natural environments, much of which eventually enters the ocean [14,15]. The continuous influx of PET into marine systems not only exacerbates persistent pollution but also imposes a selective pressure that may favor microorganisms capable of utilizing plastic as a carbon source [16]. Furthermore, many marine bacteria possess strong motility, surface adhesion, and biofilm-forming capabilities [17], enabling them to efficiently locate, attach to, and potentially degrade PET particles. Importantly, marine microorganisms have adapted to marine environmental conditions such as high salinity, complex ionic composition, and oligotrophic nutrients [18], making them particularly suitable for in situ bioremediation of plastic pollution in marine ecosystems. Therefore, marine microorganisms represent an untapped resource for discovering new PET-degrading microorganisms.

In this study, we aimed to explore the diversity of marine-derived microorganisms with PET-degrading potential and to identify novel microbial resources involved in PET biodegradation. Candidate PET-degrading microorganisms were isolated from marine samples using PET powder as the major carbon source and were subsequently characterized for PET deterioration using scanning electron microscopy (SEM) and Fourier-transform infrared (FTIR) spectroscopy. A representative PET-degrading strain, *Microbacterium aurum* SCSIO 85700, as the most efficient isolate, was further selected for comparative genomic analysis, genome annotation, and protein structure prediction to investigate the genetic basis underlying its PET-degrading potential and to identify candidate PET hydrolases and related metabolic pathways. Overall, this study not only expands the recognized diversity of marine-derived PET-degrading microorganisms but also provides microbial resources for sustainable PET bioremediation.

## 2. Materials and Methods

### 2.1. Materials and Medium

PET films (amorphous, 1 mm thickness, ES303010) were purchased from Goodfellow Ltd. (London, UK). PET powder was obtained from DuPont Co., Ltd. (Shanghai, China). Prior to use, PET films were cut into 2 cm × 2 cm squares (about 100 mg). Both PET powder and PET films were sterilized in 75% (*v*/*v*) ethanol overnight and subsequently dried at 30 °C to remove residual ethanol. Minimal salt medium (MSM) was purchased from Coolaber Technology Co., Ltd. (Beijing, China). Potato dextrose broth (PDB) and potato dextrose agar (PDA) were purchased from HuanKai Microbial Science and Technology Co., Ltd. (Guangzhou, China). Luria–Bertani (LB) medium was purchased from Sangon Biotech Co., Ltd. (Shanghai, China).

### 2.2. Isolation and Identification of Candidate PET-Degrading Marine Strains Using PET Powder as Major Carbon Source

A total of 50 marine samples, including seawater, sediments, and plastic debris, were collected from mangrove areas in Zhanjiang, China, and intertidal zones in Daya Bay, Shenzhen, China. For each sample, 1 g (wet weight) was inoculated into 10 mL of sterile natural seawater collected from Daya Bay containing glass beads and 0.1 g of PET powder and incubated at 28 °C with shaking at 150 rpm for 20 days. The incubation temperature of 28 °C was chosen because it is representative of the relatively warm seawater temperatures commonly observed in Daya Bay during summer and autumn (approximately 28–30 °C) [19], and was therefore adopted for all cultivation and degradation experiments in this study. The enriched culture was serially diluted with sterile seawater, and appropriate dilutions were spread onto MSM agar plates supplemented with 1.5% sea salt and containing PET powder as a major carbon source. Plates were incubated at 28 °C for 15–30 days. Colonies formed under these conditions, indicating the ability to utilize PET as the primary carbon and energy source, were selected for further purification. Isolates were repeatedly streaked on the same PET-containing medium until pure single colonies were obtained.

To identify the potential PET-degrading strains, genomic DNA of bacteria and fungi was extracted using the Chelex-100 method [20] and the cetyltrimethylammonium bromide (CTAB) method [21], respectively. For bacterial strains, the 16S rRNA gene was amplified with primers 27F and 1492R following the protocol described previously [22]. For fungal strains, the entire ITS region was amplified with primers ITS1 and ITS4 according to the protocol described previously [23,24]. The amplified products were purified and submitted to the Guangzhou Branch of Beijing Qingke Biotechnology Co., Ltd (Guangzhou, China). for sequencing. The verified sequences were then subjected to BLAST+ (version 2.16.0) analysis to identify the strains [25]. The information of the isolated strains was listed in Supplementary Table S1.

### 2.3. Screening and Evaluation of PET-Degrading Strains Using PET Films

Candidate PET-degrading bacteria were grown on LB agar plates at 37 °C for 7 days, and fungi were grown on PDA medium at 28 °C for 7 days. Single colonies from the respective plates were inoculated into 30 mL of MSM supplemented with 5% (*v*/*v*) LB broth (for bacteria) or 5% (*v*/*v*) PDB medium (for fungi), 1.5% sea salt, and two pieces of PET film (2 cm × 2 cm, 100 mg). Cultures were incubated at 28 °C with shaking at 200 rpm for 30 days. Every 10 days, 5 mL of culture was removed from each flask and replaced with an equal volume of fresh MSM containing 5% (*v*/*v*) LB and 1.5% sea salt to maintain nutrient availability. At the end of the incubation period, only cultures that reached OD_600_ > 0.8, indicating substantial biomass accumulation, were selected for further analysis. The PET films were then carefully retrieved from the cultures, and cells attached to the PET film surfaces were removed by washing with 75% ethanol, followed by three rinses with sterile distilled water to eliminate residual ethanol. The cleaned PET films were then dried at 30 °C and weighed to determine weight loss. In addition, the cleaned films were cut into 0.5 cm × 0.5 cm fragments for further scanning electron microscopy (SEM), Fourier-transform infrared (FTIR), and X-ray diffraction (XRD) analyses.

#### 2.3.1. SEM Analysis

For SEM analysis, PET films were sputter-coated with a 10 nm layer of gold–platinum using an ion sputter coater J20 (Supa Instrument Co., Ltd., Suzhou, China). Samples were examined under a GeminiSEM 360 SEM (Carl Zeiss Microscopy GmbH, Jena, Germany) operated at 5 kV and 100–120 μA, with images captured at 200× and 5000× magnifications.

#### 2.3.2. FTIR Analysis

FTIR analysis was performed using a Nicolet iS10 Fourier-transform infrared spectrometer (Thermo Fisher Scientific, Waltham, MA, USA) equipped with an attenuated total reflectance (ATR) accessory. Spectra were recorded at six different sites on each sample to account for surface heterogeneity, over the range of 400–4000 cm^−1^ at a resolution of 1 cm^−1^, with 44 scans averaged per sample.

#### 2.3.3. XRD Analysis

For XRD analysis, PET films were directly mounted onto the sample holder of a D8 Advance X-ray diffractometer (Bruker AXS GmbH, Karlsruhe, Germany) using Cu Kα radiation (λ = 1.5406 Å). The instrument was operated at 40 kV and 40 mA, and diffraction patterns were collected over a 2θ range of 5–90° at a scanning rate of 5° min^−1^.

### 2.4. Genome Analysis

Based on its superior PET-degrading performance, *Microbacterium aurum* SCSIO 85700 was selected for genome analysis to investigate the genetic basis of its PET degradation ability. Strain SCSIO 85700 was grown on LB medium with 3% NaCl at 28 °C with agitation for 3 days. The genomic DNA of strain SCSIO 85700 was initially extracted using the Invitrogen PureLink^®^ Genomic DNA kit (Thermo Fisher Scientific, Waltham, MA, USA). The DNA quantity and quality were tested by the NanoDrop ND-1000 Spectrophotometer (Thermo Fisher Scientific, Wilmington, DE, USA)). The DNA was purified further using the Quick-DNA Miniprep Plus kit (Zymo Research, Irvine, CA, USA). The complete genome sequencing of strain SCSIO 85700 was performed using both the PacBio Sequel II platform (Pacific Biosciences, Menlo Park, CA, USA) and the Illumina NovaSeq 6000 platform (Illumina, San Diego, CA, USA) at Biozeron Biotechnology Co., Ltd. (Shanghai, China). For PacBio sequencing, a SMRTbell library was constructed and sequenced on the PacBio Sequel II platform using three SMRT Cells 8M with 30 h movies (Sequencing Chemistry v2.0). For Illumina sequencing, a paired-end library with an insert size of approximately 450 bp was prepared and sequenced on the NovaSeq 6000 platform to generate 2 × 150 bp reads. Base calling was performed using the instrument’s default software. Raw reads were quality-filtered and adapter-trimmed using Trimmomatic (v0.36) for Illumina data [26], while PacBio reads were processed using the default quality control pipeline. Hybrid de novo assembly was performed using Unicycler (v0.4.8). The resulting genome assembly was subsequently circularized using Circlator [27], yielding a complete circular genome of 3,029,447 bp.

Gene models were identified using GeneMark (v 2.5; http://topaz.gatech.edu/GeneMark/ (accessed on 15 March 2026)) [28]. Then, all gene models were blastp-analyzed against different functional types of databases to perform functional annotation using the blastp module, including the non-redundant (NR in NCBI) database, SwissProt (http://uniprot.org (accessed on 15 March 2026)), GO (http://www.geneontology.org/ (accessed on 15 March 2026)), COG (http://www.ncbi.nlm.nih.gov/COG (accessed on 15 March 2026)), and RAST (RAST Server—RAST Annotation Server; https://rast.nmpdr.org/ (accessed on 15 March 2026)). In addition, tRNAs were identified using tRNAscan-SE (v2.0.4, http://lowelab.ucsc.edu/tRNAscan-SE (accessed on 15 March 2026)) [29], and rRNAs were determined using RNAmmer (v1.2, http://www.cbs.dtu.dk/services/RNAmmer/ (accessed on 15 March 2026)) [30]. Average nucleotide identity (ANI) between strain SCSIO 85700 and its closest strain was calculated using EzBioCloud ANI Calculator (https://www.ezbiocloud.net/ (accessed on 15 March 2026)), and the whole-genome orthologous genes in strain SCSIO 85700 and three reference strains were analyzed by Orthovenn3 (https://orthovenn3.bioinfotoolkits.net/ (accessed on 15 March 2026)).

## 3. Results and Discussion

### 3.1. Isolation of Candidate PET-Degrading Strains Using PET Powder as Major Carbon Source

**Figure 1:**
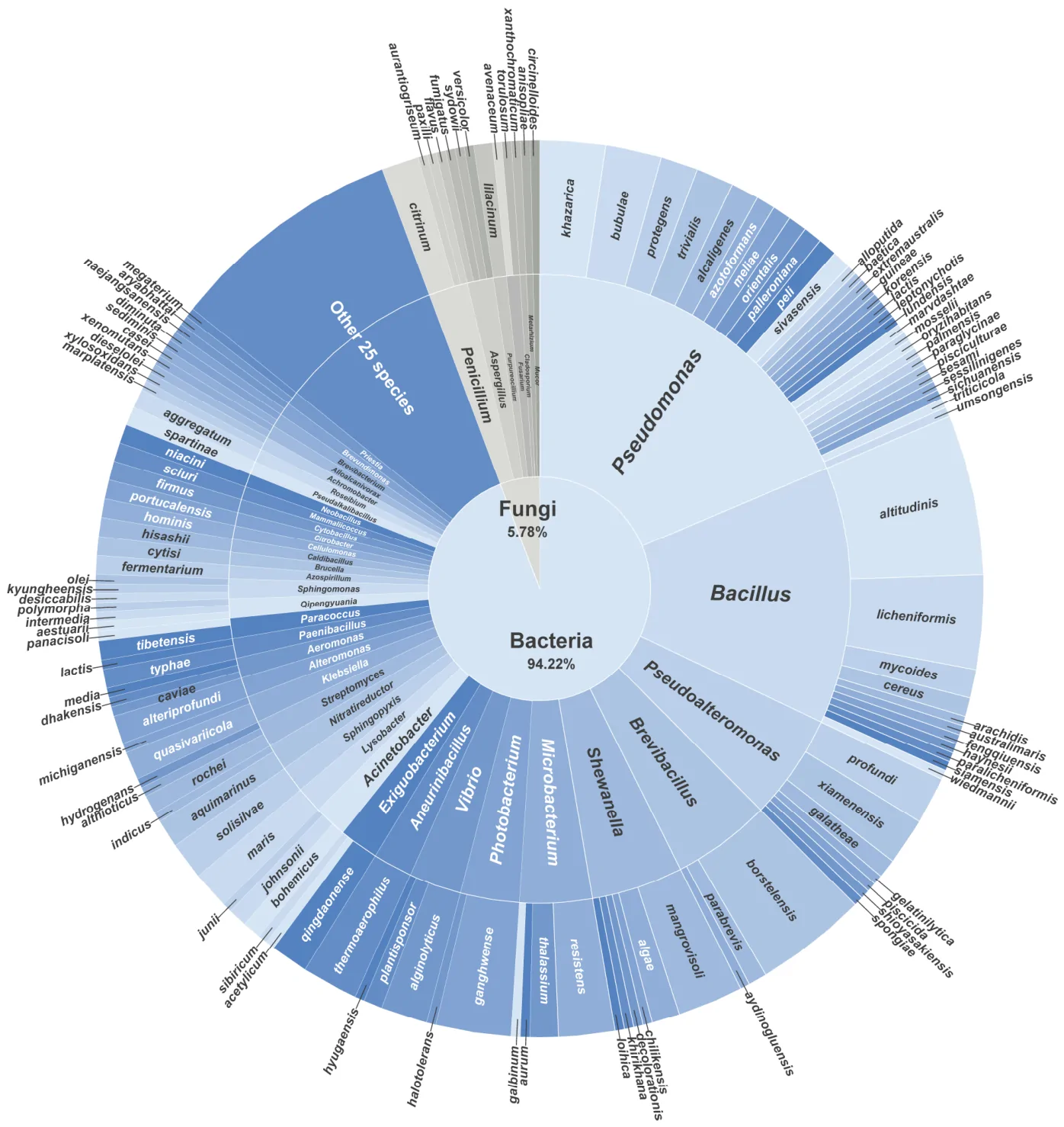
Sunburst chart of taxonomic distribution of 305 strains isolated from the medium using PET powder as a major carbon source. “Other 25 species” in the figure represents 25 low-abundance species (each < 0.34%), including *Aeribacillus composti*, *Alcaligenes faecalis* subsp. *phenolicus*, *Aquitalea magnusonii*, *Castellaniella denitrificans*, *Cupriavidus basilensis*, *Dermacoccus abyssi*, *Iodobacter fluviatilis*, *Kocuria rhizophila*, *Limibacter armeniacum*, *Lysinibacillus fusiformis*, *Mesobacillus jeotgali*, *Mesorhizobium soli*, *Microbulbifer arenaceous*, *Morganella morganii* subsp. *sibonii*, *Muricauda chongwuensis*, *Nocardioides fonticola*, *Paenibacillus typhae*, *Phenylobacterium composti*, *Providencia huaxiensis*, *Pseudooceanicola endophyticus*, *Rheinheimera pleomorphica*, *Rossellomorea aquimaris*, *Rothia marina*, *Stenotrophomonas rhizophila* and *Sinomicrobium soli*.

To explore marine environments as a source of novel PET-degrading microorganisms, 50 marine samples (seawater, sediments, and plastic debris) were collected from mangrove areas in Zhanjiang and intertidal zones in Daya Bay, Shenzhen. Following enrichment and isolation on MSM containing PET powder as the major carbon source, a total of 305 strains with PET-degrading potential were isolated and identified (Table S1 and Figure 1). Based on molecular and morphological analyses, these strains were classified into 69 genera, comprising 62 bacterial and 7 fungal genera (Supplementary Table S1). The relative frequency of each genus was calculated as the proportion of isolates belonging to that genus among the 305 identified strains. Within the 69 genera, *Pseudomonas* (18.71%), *Bacillus* (13.27%), *Pseudoalteromonas* (5.44%), *Brevibacillus* (5.10%), *Shewanella* (4.76%), *Microbacterium* (3.74%), and *Vibrio* (2.72%) were the most frequently isolated genera. Eleven additional genera, including *Streptomyces*, *Alteromonas*, *Paenibacillus*, *Brucella*, *Nitratireductor*, *Aspergillus*, *Penicillium*, *Fusarium*, *Alloalcanivorax*, *Purpureocillium*, and *Cladosporium*, were isolated less frequently (each ≤2.04%). The remaining 51 genera (Table S1), including *Aneurinibacillus*, *Exiguobacterium*, *Lysobacter*, and *Neobacillus*, were isolated at low frequency. Among the 69 genera, 18 genera have previously been reported to degrade polyester-based plastics such as PET and polyurethane (PU) or to harbor putative PET hydrolase-like genes in their genomes [31,32]. To our knowledge, the remaining 51 genera have not been previously reported in association with PET degradation. Their identification reinforces the value of marine ecosystems as a promising and underexplored resource for discovering novel PET-degrading microorganisms.

It is notable that the abundance of isolated bacteria (287 strains, 94.22%) was substantially higher than that of fungi in this study. This may be attributed to the nutrient-deficient isolation medium (MSM containing PET as the major carbon source) used here, a condition under which bacteria often outperform fungi. This phenomenon aligns with what has been described as the endurance of prolonged nutrient prevention (EPNP) phase in microbial ecology, wherein bacteria exhibit multiple adaptive starvation strategies [33]. Bacteria under extended nutrient limitation enter a long-term stationary phase, sustain slow oligotrophic growth, and express stress tolerance phenotypes, all of which enhance their survival [34]. In contrast, although fungi also possess starvation response mechanisms, their generally higher maintenance energy requirements and greater reliance on complex carbon sources often render them less persistent than bacteria under conditions of extreme nutrient scarcity [33]. It should also be noted that because the natural seawater used for primary enrichment may contain dissolved and suspended organic matter that can support microbial growth, and agar may provide an additional carbon source during subsequent isolation, the isolation and identification of the 305 strains may not necessarily be attributable to PET utilization alone. Therefore, further evaluation of PET film degradation was necessary to identify genuine degraders among the candidate strains, as described in the following section.

### 3.2. Evaluation of PET Film Degradation Performance of Candidate PET-Degrading Strains

To further assess the PET-degrading capabilities of all isolates, each strain was cultivated in MSM supplemented with 5% LB and 1.5% sea salt and containing two PET films as a major carbon source for 30 days. Because strains achieving higher biomass in PET-containing medium are more likely to possess PET-utilizing capabilities, and to reduce the number of candidates for the more time-consuming SEM, FTIR and weight-loss analyses, OD_600_ > 0.8 was used as a preliminary growth criterion for selecting isolates for subsequent PET film degradation assays. Among all isolates, 76 strains exhibited substantial growth, reaching an OD_600_ > 0.8. The PET films incubated with these 76 cultures were subsequently recovered for further analyses.

**Figure 2:**
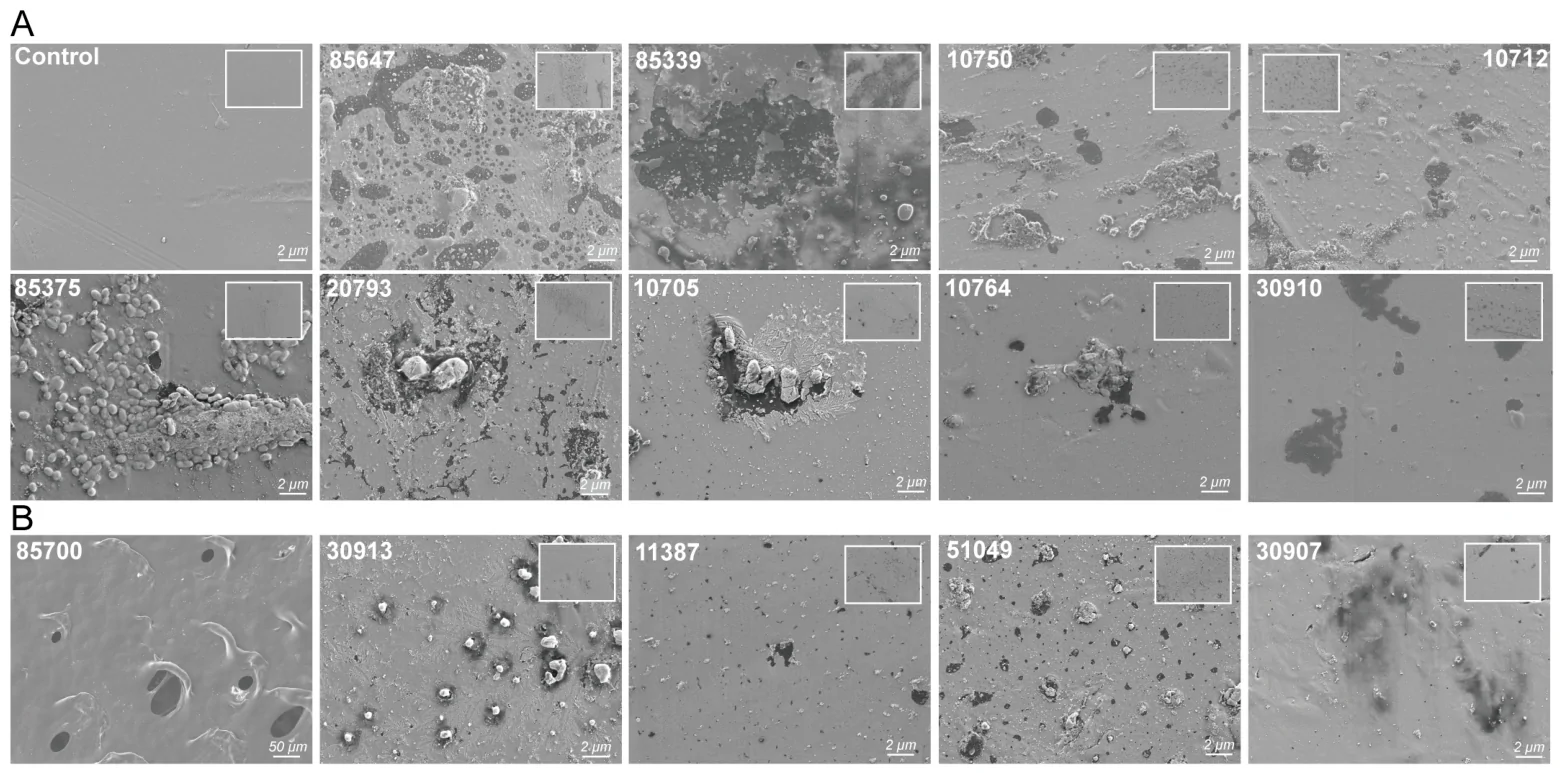
Characterization of PET degradation by PET-degrading strains using SEM. SEM images of PET films after 30 days of incubation with the 14 candidate potent degraders. (**A**) PET films showing localized surface erosion caused by strains *Paenibacillus typhae* SCSIO 85647, *Microbacterium esteraromaticum* SCSIO 85339, *Bacillus cereus* SCSIO 10750, *Microbacterium galbinum* SCSIO 10712, *Photobacterium ganghwense* SCSIO 85375, *Brevibacterium diminuta* SCSIO 20793, *Exiguobacterium qingdaonensis* SCSIO 10705, *Bacillus fengqiuensis* SCSIO 10764, and *Microbacterium jeotgali* SCSIO 30910, together with the uninoculated control. (**B**) PET films showing localized pitting caused by strains *Microbacterium aurum* SCSIO 85700, *Brevundimonas naejangsanensis* SCSIO 30913, *Brevibacterium sediminis* SCSIO 11387, *Sphingomonas olei* SCSIO 51049, and *Brevibacterium wiedmannii* SCSIO 30907. The strain numbers are indicated in the upper left or upper right corner of each micrograph. Different colors indicate the degree of PET surface alteration, with lighter and darker colors representing lower and higher degrees of surface degradation, respectively.

Structural changes in the PET films after biodegradation were analyzed using SEM. SEM observations revealed that there are 14 strains that caused varying degrees and forms of surface deterioration on PET films (Figure 2). Two distinct forms of surface deterioration were observed: surface erosion and localized pitting. Nine of these 14 strains, *Paenibacillus typhae* SCSIO 85647, *Microbacterium esteraromaticum* SCSIO 85339, *Bacillus cereus* SCSIO 10750, *Microbacterium galbinum* SCSIO 10712, *Photobacterium ganghwense* SCSIO 85375, *Brevibacterium diminuta* SCSIO 20793, *Exiguobacterium qingdaonensis* SCSIO 10705, *Bacillus fengqiuensis* SCSIO 10764, and *Microbacterium jeotgali* SCSIO 30910, caused localized surface erosion (Figure 2A). Five additional strains, *Microbacterium aurum* SCSIO 85700, *Brevundimonas naejangsanensis* SCSIO 30913, *Brevibacterium sediminis* SCSIO 11387, *Sphingomonas olei* SCSIO 51049, and *Brevibacterium wiedmannii* SCSIO 30907, caused localized, pit-like depressions on the PET surface rather than widespread surface erosion (Figure 2B). Notably, *Microbacterium aurum* SCSIO 85700 exhibited pronounced morphological changes, characterized by localized pitting with a diameter of approximately 20 µm (Figure 2B). These two forms of deterioration, surface erosion and localized pitting, may reflect differences in the spatial localization of PET-degrading enzymes. Secreted enzymes, such as *Is*PETase from *I. sakaiensis* 201-F6, can diffuse across the material surface, leading to relatively uniform erosion [10,35]. In contrast, cell-surface-anchored enzymes, such as the PET esterase from *Rhodococcus pyridinivorans* P23, restrict degradation activity to cell attachment sites, producing localized depressions or pits [36].

**Figure 3:**
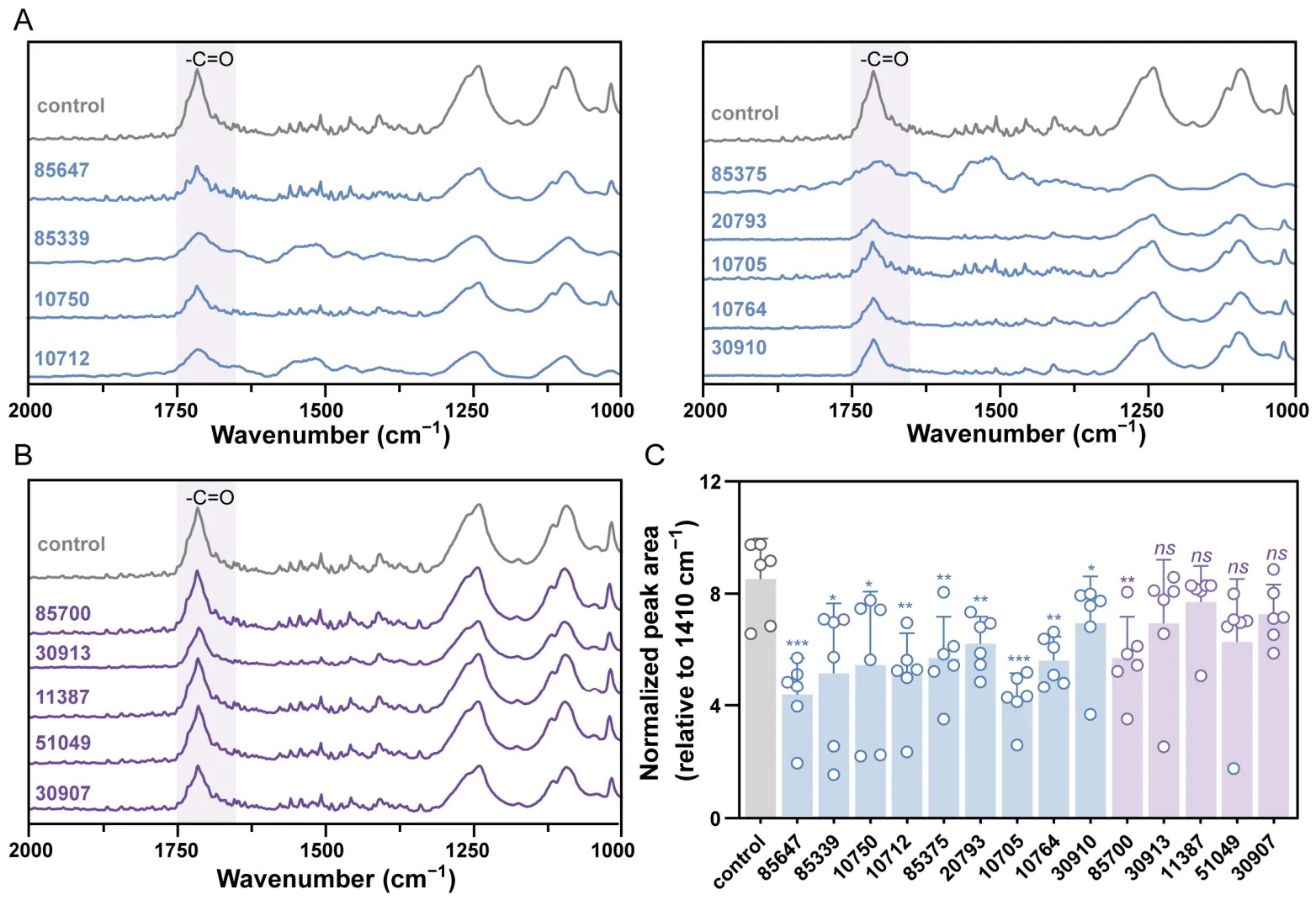
Characterization of PET degradation by PET-degrading strains using FTIR. (**A**,**B**) FTIR spectrum of PET films treated with the 14 candidate strains for 30 days. (**C**) Normalized peak area at 1710 cm^−1^. The spectra were normalized at the peak at 1410 cm^−1^ to investigate chemical changes in the peaks at 1710 cm^−1^. Statistical differences relative to the untreated control were analyzed using an unpaired *t*-test (*n* = 6, ns *p* > 0.05, * *p* < 0.05, ** *p* < 0.01, *** *p* < 0.001).

Chemical changes in the PET films after biodegradation were analyzed using FTIR. FTIR analysis also reflected differences between the two forms of surface deterioration. The characteristic absorption peak of PET at 1710 cm^−1^, corresponding to the C=O stretching of ester bonds, serves as a key indicator of degradation [37]. After baseline correction, all spectra were normalized to the least-affected band at 1410 cm^−1^ [37], and quantitative analysis of the 1710 cm^−1^ peak area was performed (Figure 3). PET films degraded by strains causing pronounced PET film surface erosion, i.e., *P. typhae* SCSIO 85647, *M. esteraromaticum* SCSIO 85339, *B. cereus* SCSIO 10750, *M. galbinum* SCSIO 10712, *P. ganghwense* SCSIO 85375, *B. diminuta* SCSIO 20793, *E. qingdaonense* SCSIO 10705, *B. fengqiuensis* SCSIO 10764, and *M. jeotgali* SCSIO 30910, showed significant reductions in the 1710 cm^−1^ peak area (Figure 3A). The relative peak area at 1710 cm^−1^ of these strains decreased by 49.46% (*P. typhae* SCSIO 85647), 39.73% (*M. esteraromaticum* SCSIO 85339), 36.29% (*B. cereus* SCSIO 10750), 40.67% (*M. galbinum* SCSIO 10712), 33.38% (*P. ganghwense* SCSIO 85375), 26.63% (*B. diminuta* SCSIO 20793), 50.43% (*E. qingdaonense* SCSIO 10705), 34.45% (*E. qingdaonense* SCSIO 10705), and 15.11% (*M. jeotgali* SCSIO 30910) (Figure 3C). For *M. aurum* SCSIO 85700, *B. naejangsanensis* SCSIO 30913, *B. sediminis* SCSIO 11387, *S. olei* SCSIO 51049, and *B. wiedmannii* SCSIO 30907, the 1710 cm^−1^ peak areas were comparable to those of these controls, suggesting negligibly detectable alteration of ester bonds on the PET film surface (Figure 3B). The relative peak area at 1710 cm^−1^ of these strains decreased by 33.38% (*M. aurum* SCSIO 85700), 18.85% (*B. naejangsanensis* SCSIO 30913), 9.91% (*B. sediminis* SCSIO 11387), 18.73% (*S. olei* SCSIO 51049), and 27.28% (*B. wiedmannii* SCSIO 30907) (Figure 3C). This observation may be attributed to their mode of deterioration: these strains formed localized pitting rather than widespread surface erosion, leaving the majority of the PET film surface chemically unaltered and thus undetectable by bulk FTIR analysis, highlighting the need to combine FTIR with SEM observations to assess surface micro-region degradation more accurately. Nevertheless, our interpretation remains tentative, as direct experimental evidence supporting these distinct modes of PET deterioration is still lacking. In particular, we have not yet characterized the secreted enzymes or other molecular determinants responsible for localized pitting versus widespread surface erosion. Future studies integrating enzymatic, proteomic, and genetic analyses will be necessary to elucidate the mechanistic basis underlying these apparently different PET degradation patterns.

**Figure 4:**
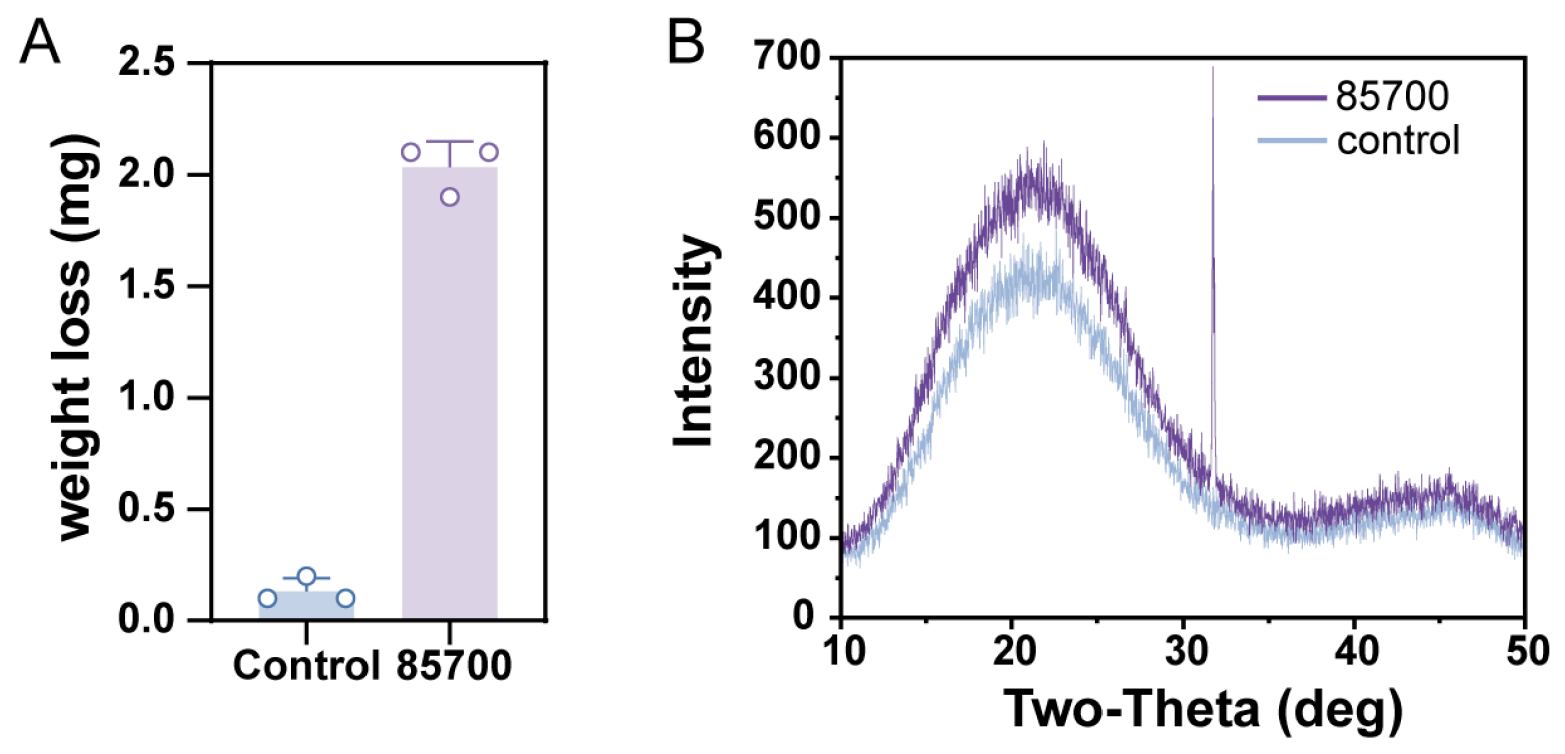
Weight loss and XRD analysis of PET films after 30 days of incubation without (control) or with SCSIO 85700. (**A**) Weight loss of PET films. (**B**) XRD patterns of PET films.

To further identify the most efficient PET degrader among the 14 verified strains, weight loss was assessed. Among them, *Microbacterium aurum* SCSIO 85700 exhibited the most potent PET-degrading activity, achieving a weight loss of 2.1 mg (2.1%) over 30 days (Figure 4A), whereas no appreciable changes were observed for the films incubated with the other strains. Meanwhile, XRD analysis was also performed to further evaluate the degradation performance of the 14 verified strains. The results showed that the PET film incubated with strain SCSIO 85700 exhibited detectable changes after 30 days of incubation, with the intensity of the diffraction peak at 2θ = 31.8° increasing from 164 to 689 and the relative crystallinity rising by 6.5% (Figure 4B), whereas no appreciable changes were observed for the films incubated with the other strains. These changes in crystallinity indicated that strain SCSIO 85700 preferentially degraded the more accessible amorphous regions of PET, whereas the crystalline regions are relatively more resistant to enzymatic hydrolysis. As the amorphous fraction is progressively removed, the proportion of the remaining crystalline domains increases, leading to an apparent increase in the relative crystallinity of the residual PET [38,39]. This phenomenon is consistent with previously reported observations on the biodegradation of PET [38,39].

It is notable that the 14 verified PET-degrading isolates belonged to eight genera and 14 distinct species, none of which, to the best of our knowledge, has been previously reported as a PET degrader. These findings reinforce the value of marine ecosystems as a promising and underexplored reservoir for discovering novel PET-degrading microorganisms. Among them, *Microbacterium aurum* strain SCSIO 85700 exhibited the strongest PET-degrading capability, achieving a weight loss of 2.1 mg (2.1%) in MSM supplemented with 1.5% sea salt to simulate seawater conditions over 30 days. Although several PET-degrading microorganisms, such as *Ideonella sakaiensis* [10,35] and *Comamonas testosteroni* [40], have been reported with high degradation efficiency under non-saline conditions, most of them originate from terrestrial environments and are adapted to non-saline conditions. In our experimental setup (LB broth medium with 3% sea salt), the representative terrestrial PET degrader, *I. sakaiensis* 201-F6, failed to grow (Supplementary Figure S1), whereas strain SCSIO 85700 exhibited clear PET degradation under the same saline conditions. This contrast indicates the potential of strain SCSIO 85700 for PET degradation in saline, marine-like environments. Based on its superior degradation performance among the 14 verified PET-degrading isolates, strain SCSIO 85700 was selected as the representative strain for subsequent investigations aimed at elucidating the genetic and biochemical bases of PET biodegradation. 

### 3.3. Genome Analysis of Strain SCSIO 85700

To investigate the genetic basis underlying the ability of strain SCSIO 85700 to degrade PET, we sequenced its complete genome using both PacBio RS and Illumina platforms. The genome of strain SCSIO 85700 comprises a single 3.02 Mb circular chromosome, with a GC content of 70.46%. Genome annotation identified 2959 protein-coding genes, alongside 45 tRNA and 44 rRNA genes. Phylogenomic analysis revealed that, at the whole-genome level, strain SCSIO 85700 showed an average nucleotide identity (ANI) value of 97.43% to *Microbacterium aurum* ASM197498v1 ^T^ [41], suggesting the classification of strain SCSIO 85700 as *Microbacterium aurum* at the species level.

**Figure 5:**
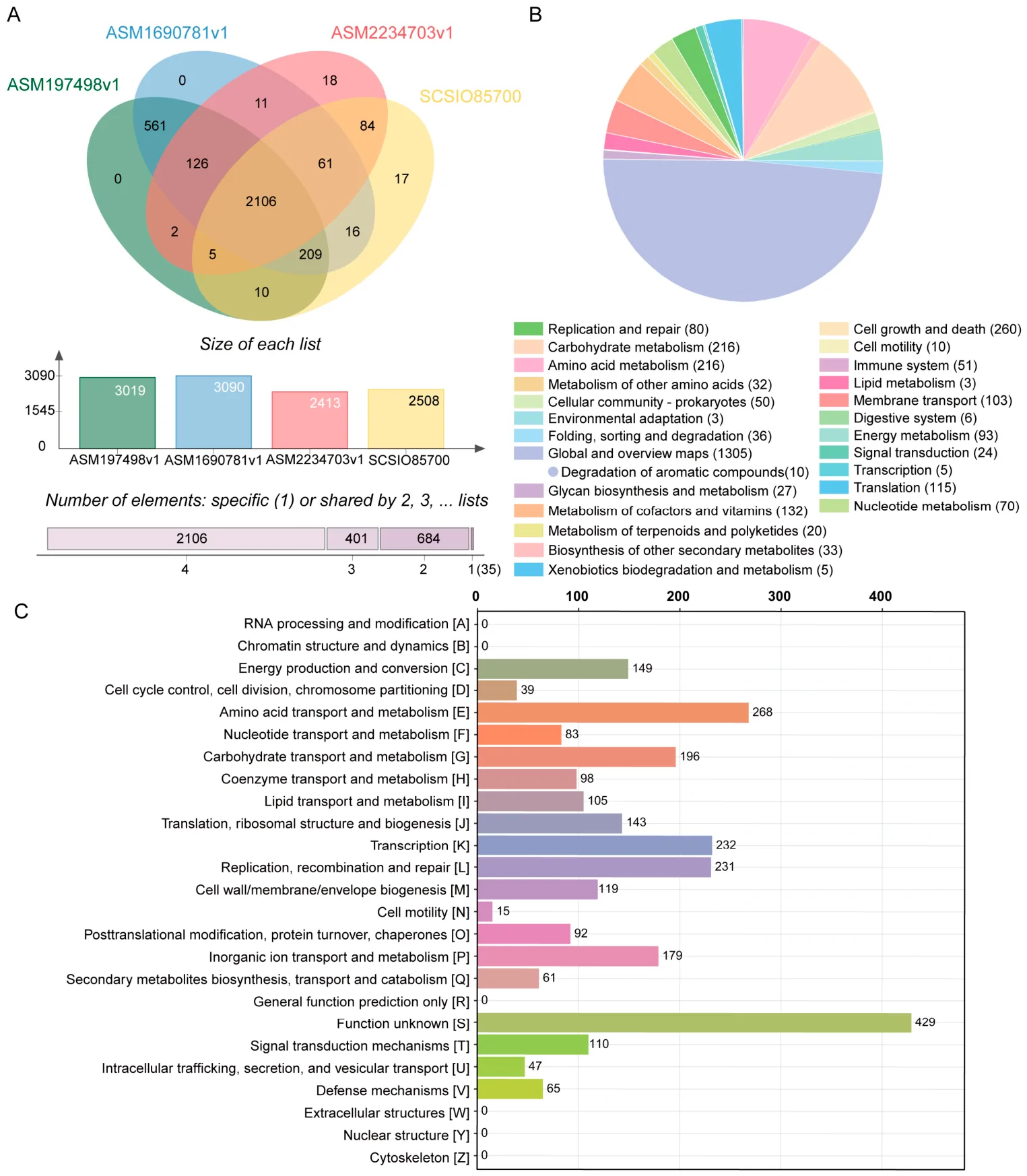
Genome analysis of SCSIO 85700. (**A**) Venn diagram of whole-genome orthologous genes in SCSIO 85700 and three reference strains. The numbers in the diagram indicate overlapped conserved genes or non-overlapped unique genes in each species. (**B**) The subsystem coverage and category distribution of the strain SCSIO 85700 genome utilizing RAST. The pie chart demonstrates the counts for each subsystem feature and the subsystem coverage. Genes for each Subsystem Category were displayed in brackets. (**C**) COG functional classification of proteins in the genome of SCSIO 85700.

To explore the genomic relatedness of strain SCSIO 85700, its genome was compared with three other published genomes of *Microbacterium aurum* strains, ASM197498v1, ASM1690781v1 and ASM2234703v1. The results are summarized in a Venn diagram (Figure 5A). A shared core genome comprising 2106 gene families was identified across all four strains, indicating a highly conserved genetic backbone within the genus. However, notable differences were observed in the accessory genome content. Strain SCSIO 85700 harbored 17 strain-specific genes that were absent from the other compared genomes. In terms of pairwise overlaps, 10 genes were uniquely shared between strain SCSIO 85700 and strain ASM197498v1, whereas strain SCSIO 85700 and strain ASM1690781v1 exhibited 16 shared genes. Additionally, 84 orthologous genes were detected exclusively between strain SCSIO 85700 and strain ASM2234703v1. These patterns suggest a conserved core genome accompanied by flexible accessory regions, potentially reflecting niche-specific adaptation and functional diversification.

There were 35 genes annotated as esterases, lipases, cutinase-like enzymes, and α/β-hydrolases in the genome of strain SCSIO 85700 (Supplementary Table S2). These enzyme families are known to catalyze the hydrolysis of ester bonds and have frequently been associated with polyester degradation. Previous studies demonstrated that *Is*PETase from *Ideonella sakaiensis* [10] and LCC [11] belong to the α/β-hydrolase superfamily and exhibit efficient PET-degrading activity. Therefore, the occurrence of homologous hydrolytic enzymes in strain SCSIO 85700 suggests a potential genetic basis for PET degradation.

The RAST annotation revealed that the strain SCSIO 85700 genome harbors genes involved in aromatic compound metabolism (Figure 5B), which may participate in the downstream utilization of PET-derived degradation products [42,43]. TPA, one of the major PET hydrolysis products, can be further metabolized through aromatic compound degradation pathways and subsequently enter central carbon metabolism [42,43]. The presence of these catabolic genes indicates that strain SCSIO 85700 may possess not only PET depolymerization capability but also the metabolic potential to assimilate PET-derived intermediates.

COG functional classification showed that genes associated with amino acid transport and metabolism, transcription, lipid transport and metabolism, and inorganic ion transport and metabolism were among the most abundant functional categories (Figure 5C). Collectively, these genomic features provide insights into the molecular mechanisms underlying PET degradation and support the potential application of strain SCSIO 85700 in plastic bioremediation.

In addition, we found multiple genetic features in the genome associated with marine environmental adaptation in strain SCSIO 85700 (Supplementary Table S3). The presence of glycine betaine ABC transporters, sodium/proton antiporters, and potassium transport systems suggests an enhanced capacity for osmotic regulation and ion homeostasis under fluctuating marine salinity conditions [44]. Meanwhile, ABC transporter systems comprising 33 related genes involved in nutrient acquisition [45], together with Sec/Tat-dependent protein export pathways, may facilitate nutrient uptake and extracellular enzyme secretion in nutrient-limited marine environments [46,47]. Genes associated with oxidative stress defense, including thioredoxin and glutathione-dependent systems, as well as DNA repair proteins such as RecA and UvrC, further indicate the ability of SCSIO 85700 to withstand environmental stresses [48].

Further studies are required to fully elucidate the molecular mechanisms underlying the assimilation of PET-derived intermediates and aromatic compounds by *Microbacterium aurum* SCSIO 85700. Future work integrating genetic manipulation, transcriptomic analyses, and biochemical characterization will help validate these predicted metabolic pathways and reveal the metabolic flexibility of this strain. Importantly, its marine origin may confer distinct ecological advantages, such as enhanced environmental robustness, adaptation to saline conditions, and the ability to function under complex marine physicochemical stresses. These characteristics underscore the potential of SCSIO 85700 as a candidate for the development of marine-based biotechnological strategies for plastic biodegradation and circular bioresource utilization.

### 3.4. Identification of Candidate PET-Degrading Enzymes and a Predicted PET Degradation Pathway in Strain SCSIO 85700

**Figure 6:**
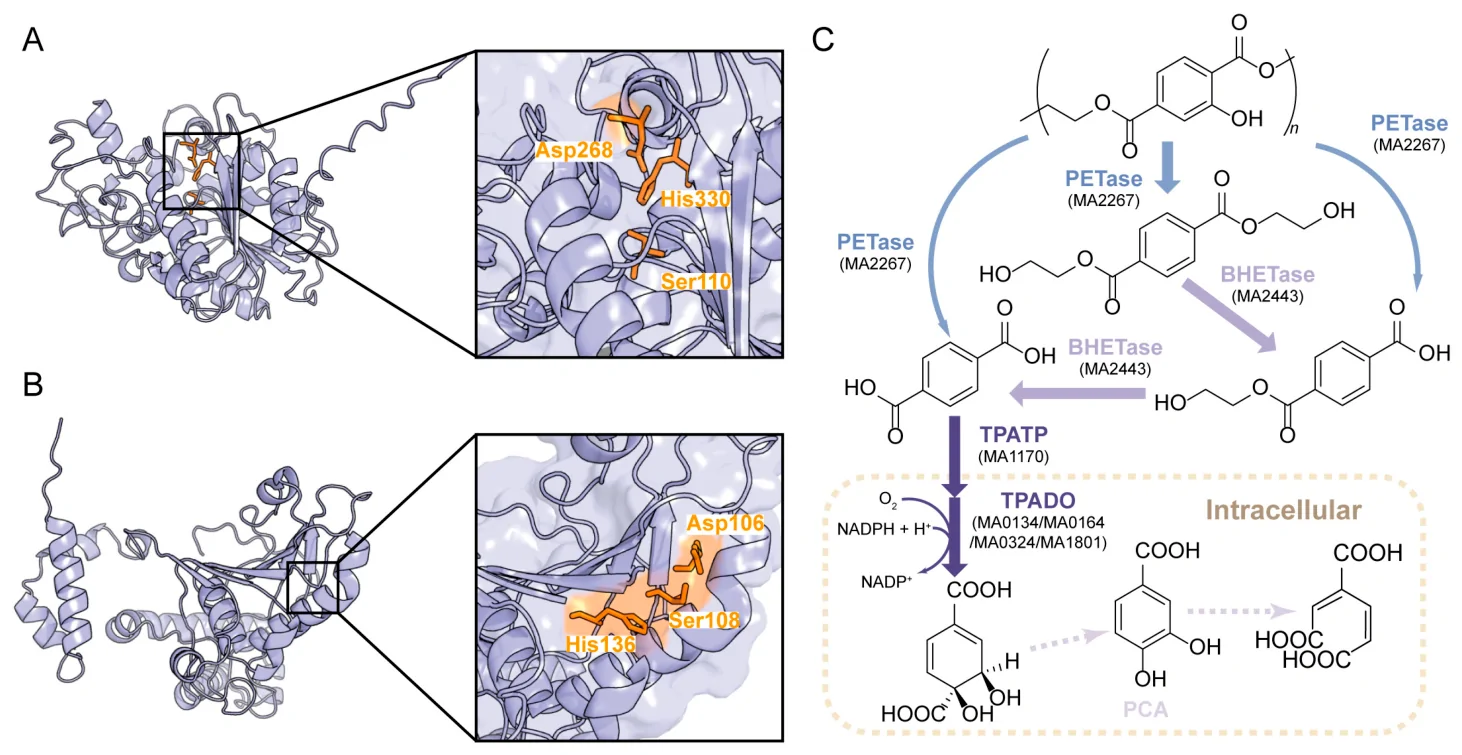
Predicted structure of candidate PET degradation protein and hypothetical degradation pathway. (**A**) Structure of MA2267. (**B**) Structure of MA2443. Active site residues are shown in orange. (**C**) Hypothetical PET degradation pathway in strain SCSIO 85700. Dashed lines indicate putative conversions without genomic evidence.

To further identify candidate proteins associated with PET degradation and metabolism in strain SCSIO 85700, we combined sequence motif screening and homology-based genome mining approaches to search its genome. Previous studies have demonstrated that characterized PET hydrolases share several conserved sequence features, including the “SMGGG” motif involved in oxyanion hole formation and a relatively conserved arrangement of catalytic triad residues [49]. Therefore, these sequence signatures were used to search for potential PET-hydrolyzing enzymes in the above mentioned 35 genes annotated as esterases, lipases, cutinase-like enzymes, and α/β-hydrolases (Supplementary Table S2), resulting in the identification of a putative PETase candidate, MA2267 (GeneID: SCSIO85700002267). Phylogenetic analysis showed that MA2267 occupied a distinct branch from the characterized PETases and other representative polyester hydrolases, suggesting that MA2267 is evolutionarily divergent from these known enzymes (Supplementary Figure S2). We also utilized 21 amino acid sequences (Supplementary Table S4) from reported enzymes that have been demonstrated to degrade polyester plastics, such as PET and PU, to search the genome. A protein annotated as carboxylesterase from strain SCSIO 85700, MA2443 (GeneID: SCSIO85700002443), exhibits sequence similarity to BHETase found in *Chryseobacterium* sp. [50], with sequence identities of approximately 42.5%, suggesting a potential role as a bis(2-hydroxyethyl) terephthalate (BHET) hydrolase, possibly catalyzing the hydrolysis of BHET to mono(2-hydroxyethyl) terephthalate (MHET) and subsequently to TPA. Subsequently, we used the algorithm AlphaFold3 to predict the structures of MA2267 and MA2443. Notably, MA2267 and MA2443 exhibited exposed, hydrophobic groove-like binding clefts (Figure 6A,B), resembling the substrate-binding architecture reported for characterized PET hydrolases [51]. Meanwhile, the active sites of enzymes MA2267 and MA2443 both consisted of a His-Asp-Ser catalytic triad located in the predicted clefts (Figure 6A,B). Such open surface topology may facilitate substrate access to the catalytic cleft and is consistent with structural features reported for polyester hydrolases [52]. While AlphaFold provides structural predictions, experimental validation is required to confirm substrate specificity and catalytic activity. Taken together, the sequence similarity, conserved catalytic residues, and predicted substrate-binding architecture support the assignment of MA2267 and MA2443 as candidate polyester hydrolases. These findings provide genomic and structural evidence supporting the PET-degrading potential of this strain.

TPA assimilation in bacteria generally relies on the coordinated action of a dedicated TPA transporter (TPATP) and TPA dioxygenase (TPADO) [10,35,42]. To gain further insight into the putative PET catabolic pathway of strain SCSIO 85700, we therefore searched its genome for previously characterized proteins involved in PET-derived aromatic compound metabolism [10,35]. Specifically, a putative TPA transporter, MA1170, was identified, which may facilitate the uptake of extracellular TPA into the cytoplasm. In addition, four genes encoding TPA 1,2-dioxygenase (TPADO), MA0134, MA0164, MA0324 and MA1801, responsible for the initial oxidation of TPA to 1,2-dihydroxy-3,5-cyclohexadiene-1,4-dicarboxylate, were identified in the genome.

Based on the genomic and structural predictions, a putative PET degradation and metabolism pathway in *Microbacterium aurum* SCSIO 85700 was proposed (Figure 6C). In this strain, the putative PET hydrolase MA2267 encoded by the chromosomal gene SCSIO85700002267 was predicted to initiate PET depolymerization by hydrolyzing PET into soluble intermediates, including BHET, MHET, and TPA. BHET could be further hydrolyzed by the putative BHETase MA2443 to generate MHET and TPA. Following PET depolymerization, TPA assimilation was predicted to occur through a dedicated uptake and degradation pathway. A putative TPA transporter, MA1170, may facilitate the incorporation of extracellular TPA into the cell, followed by oxidation mediated by TPA 1,2-dioxygenase (TPADO) to generate 1,2-dihydroxy-3,5-cyclohexadiene-1,4-dicarboxylate. This intermediate was subsequently predicted to be converted into protocatechuic acid (PCA), followed by further degradation through the β-ketoadipate pathway, ultimately yielding central metabolic intermediates that could enter the TCA cycle. Although a possible PET degradation pathway was proposed in strain SCSIO 85700, further analyses are required to clarify the complete metabolic route from PET-derived intermediates to central metabolites. Moreover, the roles of candidate genes involved in TPA transport, aromatic compound oxidation, and PCA catabolism need to be experimentally validated to fully understand the molecular mechanism underlying PET utilization. In this manner, a comprehensive PET degradation pathway of strain SCSIO 85700 can be established, providing a theoretical basis for the exploration of marine microorganisms and their enzymes for sustainable plastic biodegradation.

## 4. Conclusions

In this study, we isolated 305 candidate PET-degrading microorganisms from marine samples using a medium containing PET powder as the major carbon source. Subsequent evaluation of PET film degradation via SEM and FTIR identified 14 isolates capable of degrading PET. In addition to confirming the PET-degrading capacity of these 14 strains, we observed two distinct patterns of PET film deterioration: surface erosion and localized pitting. Notably, these 14 strains belonged to 14 distinct species, none of which, to the best of our knowledge, has been previously reported as a PET degrader. Further weight loss and XRD analysis revealed that *Microbacterium aurum* SCSIO 85700 emerged as the most efficient isolate, achieving a weight loss of 2.1 mg (2.1%) and a 6.5% increase in relative crystallinity over 30 days. To elucidate the genetic basis of its PET-degrading capacity, we performed comparative genomic analysis, genome annotation, and structural prediction to identify putative PET hydrolases and related metabolic pathways in strain SCSIO 85700. Genome analysis revealed a broad repertoire of genes associated with polyester degradation. Further genome mining and structural modeling identified two candidate PET hydrolases, MA2267 and MA2443, which possess conserved His–Asp–Ser catalytic triads and exposed substrate-binding clefts characteristic of polyester-degrading enzymes. In addition, genomic analysis uncovered a putative TPA assimilation pathway involving the TPA transporter MA1170 and multiple TPA 1,2-dioxygenases, suggesting that strain SCSIO 85700 may utilize PET-derived aromatic intermediates through downstream degradation pathways. The genome also harbors multiple adaptive traits associated with marine environments, including osmotic regulation, nutrient acquisition, stress resistance, and protein secretion systems, which may contribute to its ecological fitness under nutrient-limited marine conditions.

Overall, these findings suggest that marine environments may harbor a broader and more diverse repertoire of PET-degrading microorganisms than currently recognized. The distinct patterns of PET surface deterioration observed among the isolates further indicate that microbial PET degradation can occur through different modes of surface attack rather than a single uniform degradation process. In the case of *Microbacterium aurum* SCSIO 85700, the coexistence of putative PET-hydrolyzing enzymes, downstream aromatic compound assimilation pathways, and multiple marine-adaptive traits suggests a potentially integrated strategy for PET transformation and utilization under marine conditions. Thus, marine-derived microorganisms represent not only a valuable source of novel PET-degrading enzymes but also promising candidates for understanding and developing environmentally relevant strategies for PET biodegradation and bioremediation. Further biochemical and genetic studies will be necessary to experimentally validate the functions of the candidate enzymes and clarify their contribution to PET degradation.

## Data Availability

The original contributions presented in this study are included in the article. Further inquiries can be directed to the corresponding authors.

## Supplementary Materials

The following supporting information can be downloaded at: https://www.mdpi.com/article/10.3390/microorganisms14081804/s1, Table S1: Taxonomic of 305 strains isolated by using PET powder as sole carbon source; Table S2: Putative esterase-, lipase-, cutinase-like enzyme-, and α/β-hydrolase-encoding genes identified in the genome of *Microbacterium aurum* SCSIO 85700; Table S3: Representative genes associated with marine environmental adaptation in *Microbacterium aurum* SCSIO 85700; Table S4: Reported plastic-degrading enzymes used as query proteins for homology searches in the genome of *Microbacterium aurum* SCSIO 85700; Figure S1: Growth of *Microbacterium aurum* SCSIO 85700 and *Ideonella sakaiensis* in LB medium at different salinities after 7 Days; Figure S2: Phylogenetic analysis of the putative PET hydrolase MA2267.

## Abbreviations

The following abbreviations are used in this manuscript:

PET	Polyethylene Terephthalate
TPA	Terephthalic Acid
EG	Ethylene Glycol
MHET	Mono(2-Hydroxyethyl) Terephthalate
LCC	Leaf-branch Compost Cutinase
MSM	Minimal Salt Medium
PDB	Potato Dextrose Broth
PDA	Potato Dextrose Agar
CTAB	Cetyltrimethylammonium Bromide
LB	Luria–Bertani
SEM	Scanning Electron Microscopy
FTIR	Fourier-Transform Infrared
ATR	Attenuated Total Reflectance
XRD	X-ray Diffraction
PU	Polyurethane
EPNP	Endurance of Prolonged Nutrient Prevention
BHETase	Bis(2-Hydroxyethyl) Terephthalate Hydrolase
BHET	Bis(2-Hydroxyethyl) Terephthalate
TPATP	TPA Transporter
TPADO	TPA Dioxygenase
PCA	Protocatechuic Acid
