# Supplementary material for: Isolation of Marine-Derived Microorganisms for PET Biodegradation
Source: Microorganisms. 2026 Aug 16;14(8):1804. doi: 10.3390/microorganisms14081804 (PMC13515367; doi:10.3390/microorganisms14081804)
Supplement: Supplementary file 1 [file microorganisms-14-01804-s001.zip › microorganisms-4475416-supplementary.pdf]

Supplementary materials

Isolation of Marine-Derived microorganisms for PET Biodegradation

Shijing Deng [1,2,3,†], Qiaoqiao Guo [1,†], Yunhe An [1,3], Yuqing Liu [1,3], Jianping Yin [1], Songbiao Shi [1], Tingbiao Wu [1], Chenlu Gu [1,2], Xinpeng Tian [1,*] and Qinglian Li [1,2,*]

[1] State Key Laboratory of Tropical Oceanography, Guangdong Key Laboratory of Marine Materia Medica, South China Sea Institute of Oceanology, Chinese Academy of Sciences, Guangzhou 510301, China; dengshijing23@mails.ucas.ac.cn (S. D.); guoqiaoqiao@scsio.ac.cn (Q. G.)； anyunhe24@mails.ucas.ac.cn (Y. A.); liuyuqing232@mails.ucas.ac.cn (Y. L.); yjp@scsio.ac.cn (J. Y.); sbshi@scsio.ac.cn (S. S.); wutingbiao@szbl.ac.cn (T. W.); cheryl_chenlu@foxmail.com (C. G.)
[2] Department of Marine Pharmacy, School of Life Science and Biopharmaceutics, Guangdong Pharmaceutical University, Guangzhou 510006, China
[3] University of Chinese Academy of Sciences, Beijing 100049, China
* Correspondence: liql@scsio.ac.cn (Q. L.); xinpengtian@scsio.ac.cn (X. T.).
† These authors contributed equally to this work.

Supplementary materials:

Table S1 Taxonomic of 305 strains isolated by using PET powder as sole carbon source.

Bacteria					
genus	species	Strain number	genus	species	Strain number
Pseudomonas	*khazarica*	85332	*Microbacterium*	*esteraromaticum*	85336
Pseudomonas	*khazarica*	85357	*Microbacterium*	*esteraromaticum*	85337
Pseudomonas	*khazarica*	85377	*Microbacterium*	*esteraromaticum*	85339
Pseudomonas	*khazarica*	85422	*Microbacterium*	*esteraromaticum*	85345
Pseudomonas	*khazarica*	85423	*Microbacterium*	*esteraromaticum*	85355
Pseudomonas	*khazarica*	85428	*Microbacterium*	*resistens*	85346
Pseudomonas	*khazarica*	85723	*Microbacterium*	*resistens*	85352
Pseudomonas	*bubulae*	85014	*Microbacterium*	*resistens*	85369
Pseudomonas	*bubulae*	85053	*Microbacterium*	*thalassium*	85421
Pseudomonas	*bubulae*	85071	*Microbacterium*	*thalassium*	85433
Pseudomonas	*bubulae*	85087	*Microbacterium*	*aurum*	85674
Pseudomonas	*bubulae*	85095	*Microbacterium*	*aurum*	85700
Pseudomonas	*bubulae*	85152	*Microbacterium*	*galbinum*	10712
Pseudomonas	*protegens*	85023	*Microbacterium*	*ginsengisoli*	10792
Pseudomonas	*protegens*	85119	*Photobacterium*	*ganghwense*	85349
Pseudomonas	*protegens*	85143	*Photobacterium*	*ganghwense*	85358
Pseudomonas	*protegens*	85003	*Photobacterium*	*ganghwense*	85359
Pseudomonas	*protegens*	85158	*Photobacterium*	*ganghwense*	85407
Pseudomonas	*trivialis*	85004	*Photobacterium*	*ganghwense*	85516
Pseudomonas	*trivialis*	85009	*Photobacterium*	*ganghwense*	85372
Pseudomonas	*trivialis*	85051	*Photobacterium*	*ganghwense*	85374
Pseudomonas	*trivialis*	85052	*Photobacterium*	*ganghwense*	85375
Pseudomonas	*alcaligenes*	85017	*Photobacterium*	*halotolerans*	85419

Pseudomonas	*alcaligenes*	85112	*Vibrio*	*alginolyticus*	85127
Pseudomonas	*alcaligenes*	85725	*Vibrio*	*alginolyticus*	85399
Pseudomonas	*alcaligenes*	85429	*Vibrio*	*alginolyticus*	85430
Pseudomonas	*azotoformans*	85011	*Vibrio*	*alginolyticus*	85431
Pseudomonas	*azotoformans*	85149	*Vibrio*	*alginolyticus*	85439
Pseudomonas	*meliae*	85148	*Vibrio*	*plantisponsor*	85295
Pseudomonas	*meliae*	85160	*Vibrio*	*plantisponsor*	85722
Pseudomonas	*orientalis*	85001	*Vibrio*	*hyugaensis*	85413
Pseudomonas	*orientalis*	85006	*Aneurinibacillus*	*thermoaerophilus*	85245
Pseudomonas	*palleroniana*	85016	*Aneurinibacillus*	*thermoaerophilus*	85247
Pseudomonas	*palleroniana*	85034	*Aneurinibacillus*	*thermoaerophilus*	85251
Pseudomonas	*peli*	85661	*Aneurinibacillus*	*thermoaerophilus*	85255
Pseudomonas	*peli*	85663	*Aneurinibacillus*	*thermoaerophilus*	85263
Pseudomonas	*sivasensis*	85035	*Aneurinibacillus*	*thermoaerophilus*	85268
Pseudomonas	*sivasensis*	85091	*Exiguobacterium*	*qingdaonense*	10705
Pseudomonas	*alloputida*	85391	*Exiguobacterium*	*qingdaonense*	85720
Pseudomonas	*baetica*	85038	*Exiguobacterium*	*qingdaonense*	85729
Pseudomonas	*extremaustralis*	85138	*Exiguobacterium*	*qingdaonense*	85412
Pseudomonas	*guineae*	85662	*Exiguobacterium*	*acetylicum*	85008
Pseudomonas	*koreensis*	85090	*Exiguobacterium*	*sibiricum*	85125
Pseudomonas	*lactis*	85150	*Acinetobacter*	*bohemicus*	85123
Pseudomonas	*leptonychotis*	85664	*Acinetobacter*	*bohemicus*	85124
Pseudomonas	*lundensis*	85012	*Acinetobacter*	*johnsonii*	85335
Pseudomonas	*marvdashtae*	85153	*Acinetobacter*	*johnsonii*	85140
Pseudomonas	*mosselii*	85294	*Acinetobacter*	*junii*	85518
Pseudomonas	*oryzihabitans*	85197	*Lysobacter*	*maris*	85340
Pseudomonas	*palmensis*	85002	*Lysobacter*	*maris*	85366
Pseudomonas	*paraglycinae*	85050	*Lysobacter*	*maris*	85368
Pseudomonas	*pisciculturae*	85122	*Lysobacter*	*maris*	85417
Pseudomonas	*sesami*	85147	*Sphingopyxis*	*solisilvae*	85666
Pseudomonas	*sessilinigenes*	85157	*Sphingopyxis*	*solisilvae*	85677
Pseudomonas	*sichuanensis*	85161	*Sphingopyxis*	*solisilvae*	85678
Pseudomonas	*triticicola*	85021	*Sphingopyxis*	*solisilvae*	85681
Pseudomonas	*umsongensis*	85151	*Klebsiella*	*quasivariicola*	85040
Bacillus	*altitudinis*	85010	*Klebsiella*	*quasivariicola*	85426
Bacillus	*altitudinis*	85018	*Klebsiella*	*quasivariicola*	85503
Bacillus	*altitudinis*	85020	*Klebsiella*	*michiganensis*	85506
Bacillus	*altitudinis*	85077	*Nitratireductor*	*aquimarinus*	85338
Bacillus	*altitudinis*	85310	*Nitratireductor*	*aquimarinus*	85344

Bacillus	*altitudinis*	85315	*Nitratireductor*	*aquimarinus*	85348
Bacillus	*altitudinis*	85317	*Nitratireductor*	*indicus*	85370
Bacillus	*altitudinis*	85324	*Streptomyces*	*rochei*	85504
Bacillus	*altitudinis*	85331	*Streptomyces*	*rochei*	85514
Bacillus	*altitudinis*	85334	*Streptomyces*	*althioticus*	85515
Bacillus	*altitudinis*	85378	*Streptomyces*	*hydrogenans*	85350
Bacillus	*altitudinis*	85408	*Alteromonas*	*alteriprofundi*	85115
Bacillus	*altitudinis*	85425	*Alteromonas*	*alteriprofundi*	85432
Bacillus	*altitudinis*	85665	*Alteromonas*	*alteriprofundi*	85434
Bacillus	*altitudinis*	85675	*Paenibacillus*	*typhae*	85647
Bacillus	*altitudinis*	85676	*Paenibacillus*	*typhae*	85649
Bacillus	*altitudinis*	85701	*Paenibacillus*	*lactis*	85650
Bacillus	*altitudinis*	85703	*Paracoccus*	*tibetensis*	85363
Bacillus	*altitudinis*	85686	*Paracoccus*	*tibetensis*	85623
Bacillus	*licheniformis*	85092	*Paracoccus*	*panacisoli*	85073
Bacillus	*licheniformis*	85093	*Aeromonas*	*caviae*	85174
Bacillus	*licheniformis*	85103	*Aeromonas*	*dhakensis*	85172
Bacillus	*licheniformis*	85107	*Aeromonas*	*media*	85100
Bacillus	*licheniformis*	85116	*Sphingomonas*	*desiccabilis*	85187
Bacillus	*licheniformis*	85154	*Sphingomonas*	*kyungheensis*	85669
Bacillus	*licheniformis*	85170	*Sphingomonas*	*olei*	51049
Bacillus	*licheniformis*	85203	*Azospirillum*	*fermentarium*	85680
Bacillus	*licheniformis*	85252	*Azospirillum*	*fermentarium*	85683
Bacillus	*licheniformis*	85262	*Brucella*	*cytisi*	85367
Bacillus	*mycoides*	85104	*Brucella*	*cytisi*	85418
Bacillus	*mycoides*	85144	*Caldibacillus*	*hisashii*	85249
Bacillus	*mycoides*	85179	*Caldibacillus*	*hisashii*	85270
Bacillus	*mycoides*	85145	*Cellulomonas*	*hominis*	85702
Bacillus	*cereus*	10750	*Cellulomonas*	*hominis*	85709
Bacillus	*cereus*	85668	*Citrobacter*	*portucalensis*	85173
Bacillus	*arachidis*	85667	*Citrobacter*	*portucalensis*	85175
Bacillus	*australimaris*	85076	*Cytobacillus*	*firmus*	85364
Bacillus	*fengqiuensis*	10764	*Cytobacillus*	*firmus*	85365
Bacillus	*haynesii*	85130	*Mammaliicoccus*	*sciuri*	85057
Bacillus	*paralicheniformis*	85699	*Mammaliicoccus*	*sciuri*	85058
Bacillus	*siamensis*	85624	*Neobacillus*	*niacini*	85672
Bacillus	*wiedmannii*	30907	*Neobacillus*	*niacini*	85673
Pseudoalteromonas	*profundi*	85101	*Pseudalkalibacillus*	*spartinae*	85298
Pseudoalteromonas	*profundi*	85313	*Pseudalkalibacillus*	*spartinae*	85299
Pseudoalteromonas	*profundi*	85329	*Roseibium*	*aggregatum*	10911
Pseudoalteromonas	*profundi*	85427	*Roseibium*	*aggregatum*	85296
Pseudoalteromonas	*profundi*	85726	*Achromobacter*	*marplatensis*	85505
Pseudoalteromonas	*xiamenensis*	85342	*Achromobacter*	*xylosoxidans*	85395

Pseudoalteromonas	*xiamenensis*	85387	*Alloalcanivorax*	*dieselolei*	85438
Pseudoalteromonas	*xiamenensis*	85405	*Alloalcanivorax*	*xenomutans*	85393
Pseudoalteromonas	*galatheae*	85301	*Brevibacterium*	*casei*	85117
Pseudoalteromonas	*galatheae*	85322	*Brevibacterium*	*sediminis*	11387
Pseudoalteromonas	*gelatinilytica*	85354	*Brevundimonas*	*diminuta*	20793
Pseudoalteromonas	*piscicida*	85386	*Brevundimonas*	*naejangsanensis*	30913
Pseudoalteromonas	*shioyasakiensis*	85406	*Priestia*	*aryabhattai*	85721
Pseudoalteromonas	*spongiae*	85319	*Priestia*	*megaterium*	85078
Brevibacillus	*borstelensis*	85204	*Aeribacillus*	*composti*	85264
Brevibacillus	*borstelensis*	85243	*Alcaligenes*	*faecalis subsp. phenolicus*	85176
Brevibacillus	*borstelensis*	85248	*Aquitalea*	*magnusonii*	85129
Brevibacillus	*borstelensis*	85250	*Castellaniella*	*denitrificans*	85341
Brevibacillus	*borstelensis*	85254	*Cupriavidus*	*basilensis*	85133
Brevibacillus	*borstelensis*	85258	*Dermacoccus*	*abyssi*	85074
Brevibacillus	*borstelensis*	85260	*Iodobacter*	*fluviatilis*	85126
Brevibacillus	*borstelensis*	85261	*Kocuria*	*rhizophila*	85059
Brevibacillus	*borstelensis*	85265	*Limibacter*	*armeniacum*	85353
Brevibacillus	*borstelensis*	85266	*Lysinibacillus*	*fusiformis*	85097
Brevibacillus	*borstelensis*	85269	*Mesobacillus*	*jeotgali*	30910
Brevibacillus	*parabrevis*	85682	*Mesorhizobium*	*soli*	85648
Brevibacillus	*parabrevis*	85684	*Microbulbifer*	*arenaceous*	85420
Brevibacillus	*aydinogluensis*	85256	*Morganella*	*morganii subsp. sibonii*	85177
Shewanella	*mangrovisoli*	85379	*Muricauda*	*chongwuensis*	85283
Shewanella	*mangrovisoli*	85400	*Nocardioides*	*fonticola*	85271
Shewanella	*mangrovisoli*	85401	*Pantoea*	*cypripedii*	85383
Shewanella	*mangrovisoli*	85502	*Phenylobacterium*	*composti*	85671
Shewanella	*mangrovisoli*	85513	*Providencia*	*huaxiensis*	85159
Shewanella	*mangrovisoli*	85517	*Pseudooceanicola*	*endophyticus*	85351
Shewanella	*mangrovisoli*	85519	*Rheinheimera*	*pleomorphica*	85389
Shewanella	*algae*	85307	*Rossellomorea*	*aquimaris*	85710
Shewanella	*algae*	85039	*Rothia*	*marina*	85060
Shewanella	*algae*	85424	*Sinomicrobium*	*soli*	85404
Shewanella	*chilikensis*	85325	*Stenotrophomonas*	*rhizophila*	85079
Shewanella	*decolorationis*	85330	*Qipengyuania*	*aestuarii*	85084
Shewanella	*khirikhana*	85297	*Qipengyuania*	*intermedia*	85007
Shewanella	*loihica*	85303	*Qipengyuania*	*polymorpha*	85083
Fungi					

genus	species	Strain number	genus	species	Strain number
Penicillium	*citrinum*	85200	*Aspergillus*	*flavus*	85188
Penicillium	*citrinum*	85102	*Aspergillus*	*fumigatus*	85195
Penicillium	*citrinum*	85188	*Aspergillus*	*sydowii*	85362
Penicillium	*citrinum*	85098	*Aspergillus*	*versicolor*	85409
Penicillium	*citrinum*	85185	*Purpureocillium*	*lilacinum*	85403
Penicillium	*aurantiogriseum*	85094	*Purpureocillium*	*lilacinum*	85416
Penicillium	*paxilli*	85501	*Fusarium*	*avenaceum*	85139
Cladosporium	*xanthochromaticum*	85292	*Fusarium*	*torulosum*	85146
Metarhizium	*anisopliae*	85128	*Mucor*	*circinelloides*	85189

Table S2 Putative esterase-, lipase-, cutinase-like enzyme-, and α/β-hydrolase-encoding genes identified in the genome of *Microbacterium aurum* SCSIO 85700.

Enzyme	Gene ID	Annotation
MA0128	SCSIO85700000128	alpha/beta hydrolase
MA0127	SCSIO85700000127	alpha/beta fold hydrolase
MA2281	SCSIO85700002281	alpha/beta hydrolase
MA1497	SCSIO85700001497	alpha/beta hydrolase
MA1601	SCSIO85700001601	alpha/beta hydrolase
MA1343	SCSIO85700001343	alpha/beta hydrolase
MA0366	SCSIO85700000366	esterase
MA1913	SCSIO85700001913	alpha/beta hydrolase
MA0744	SCSIO85700000744	alpha/beta hydrolase
MA1414	SCSIO85700001414	acyl-CoA thioesterase II
MA2442	SCSIO85700002442	alpha/beta hydrolase
MA0501	SCSIO85700000501	glycerophosphodiester phosphodiesterase
MA1128	SCSIO85700001128	alpha/beta hydrolase
MA2204	SCSIO85700002204	alpha/beta hydrolase
MA1121	SCSIO85700001121	alpha/beta hydrolase
MA1415	SCSIO85700001415	4-hydroxybenzoyl-CoA thioesterase
MA1159	SCSIO85700001159	glycerophosphodiester phosphodiesterase
MA0188	SCSIO85700000188	acyl esterase
MA20902	SCSIO85700002902	alpha/beta hydrolase
MA1169	SCSIO85700001169	GDSL family lipase
MA2450	SCSIO85700002450	lysophospholipase
MA2581	SCSIO85700002581	alpha/beta hydrolase
MA2443	SCSIO85700002443	carboxylesterase
MA1455	SCSIO85700001455	alpha/beta hydrolase
MA0765	SCSIO85700000765	putative GDSL-like Lipase/Acylhydrolase
MA1174	SCSIO85700001174	thioesterase
MA1921	SCSIO85700001921	alpha/beta hydrolase
MA1880	SCSIO85700001880	alpha/beta hydrolase
MA2267	SCSIO85700002267	alpha/beta hydrolase
MA2479	SCSIO85700002479	4-hydroxybenzoyl-CoA thioesterase
MA0847	SCSIO85700000847	phosphodiesterase
MA2221	SCSIO85700002221	alpha/beta hydrolase
MA1724	SCSIO85700001724	alpha/beta hydrolase
MA1252	SCSIO85700001252	alpha/beta hydrolase
MA1782	SCSIO85700001781	tetrameric acyl-CoA thioesterase

Table S3. Representative genes associated with marine environmental adaptation in *Microbacterium aurum* SCSIO 85700.

Functional category	Representative genes/proteins	Representative locus tag(s)	Putative role
Osmotic stress adaptation	ABC-type glycine betaine transporter	SCSIO85700002095, SCSIO85700000068	Uptake of compatible solutes for osmoprotection
	BCCT family betaine/carnitine/choline transporter	SCSIO85700002695	Compatible solute transport
	Na+/H+ antiporters (Mnh system)	SCSIO85700000044, SCSIO85700000047, SCSIO85700000049, SCSIO85700001785, SCSIO85700002402–02408	Sodium extrusion, pH regulation and ion homeostasis
	Potassium transporter (TrkA)	SCSIO85700002265	Potassium uptake and osmotic balance
Nutrient acquisition	ABC transporter systems	33 representative ABC transporter-related genes identified (e.g., SCSIO85700001607, SCSIO85700001608, SCSIO85700001833, SCSIO85700001992, SCSIO85700002109, SCSIO85700002135, SCSIO85700002185, SCSIO85700002934)	Uptake of amino acids, carbohydrates, phosphate, peptides and other nutrients
Protein secretion	Sec-dependent protein export system	SecA (SCSIO85700000702, SCSIO85700002316), SecDF (SCSIO85700001229, SCSIO85700001230), SecY (SCSIO85700000441), SecE (SCSIO85700002154), SecG (SCSIO85700001086)	General secretion of proteins including extracellular enzymes
	Tat-dependent protein export system	TatB (SCSIO85700001686), TatC (SCSIO85700001155)	Export of folded proteins
Oxidative stress defense	Thioredoxin system	SCSIO85700000203, SCSIO85700000210, SCSIO85700000445, SCSIO85700000882, SCSIO85700001991, SCSIO85700002202, SCSIO85700002317, SCSIO85700002949, DSBA-like thioredoxin proteins	Maintenance of intracellular redox homeostasis

		(SCSIO85700001326, SCSIO85700002855)	
	Glutathione-dependent system	Glutathione peroxidase (SCSIO85700001324), Lactoylglutathione lyase (SCSIO85700001075, SCSIO85700002620)	Protection against oxidative stress
DNA repair	Homologous recombination and nucleotide excision repair	RecA-related proteins (SCSIO85700000895, SCSIO85700000905, SCSIO85700001459, SCSIO85700000294); UvrB (SCSIO85700001074); UvrC (SCSIO85700001079)	DNA repair and genome stability

Table S4. Reported plastic-degrading enzymes used as query proteins for homology searches in the genome of *Microbacterium aurum* SCSIO 85700.

Enzyme	Accession number	Origin	Plastic type
*Tf*Cut2	ADV9252 7.1	*Thermobifida cellulosilytica*	PEF, PET, PU
*Is*PETase	GAP38373.1	*Ideonella sakaiensis*	PET
LCC	AEV21261.1	*Uncultured bacterium*	PEF, PET, PU
MHETase	GAP38373.1	*Ideonella sakaiensis*	PET
*Tf*H	WP011291330.1	*Thermobifida fusca*	PET
BHETase	WP263602248.1	*Chryseobacterium sp.*	PET
urethanase	WBR49957.1	*uncultured bacterium*	PU
LipasePueA	AAD22743.1	*Pseudomonas chlororaphis*	PU
LipasePueB	AAF01331.1	*Pseudomonas chlororaphis*	PU
polyurethane esterase	BAA76305.1	*Delftia acidovorans*	PU
CpCut1	ON037531	*Cladosporium sp.*	PU
Tcur 1278	ACY96861.1	*Thermomonospora curvata*	PET, PU
Tcur0390	CDN67546.1	*Thermomonospora curvata*	PET, PU
CalB	P41365.1	*Moesziomyces antarcticus*	PET, PU
EsterasePulA	AAF66684.1	*Pseudomonas fluorescens*	PU
PueA	XXI88264.1	*Pseudomonas protegens*	PU
PueB	XXI88267.1	*Pseudomonas protegens*	PU
UMG-SP-1	9GIZ_B	*uncultured bacterium*	PU
UMG-SP-2	WBR49957.1	*uncultured bacterium*	PU
UMG-SP-3	WBR49958.1	*uncultured bacterium*	PU
Amidase	SCY08442.1	*Rhodococcus erythropolis*	PU

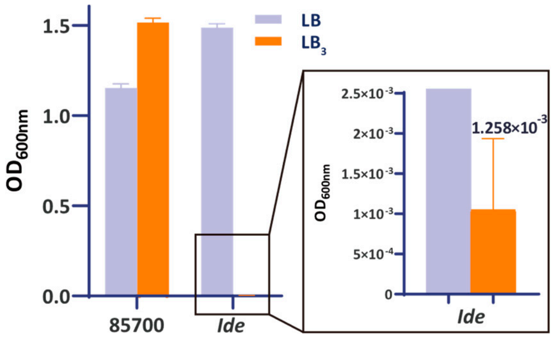

Figure S1. Growth of *Microbacterium aurum* SCSIO 85700 and *Ideonella sakaiensis* in LB medium at different salinities after 7 Days. LB_3, LB medium supplemented with 3% NaCl. *Ide*, *Ideonella sakaiensis*. Bacterial growth was evaluated by measuring the optical density at 600 nm (OD_{600nm}). The growth of *I. sakaiensis* was markedly restricted in LB_3 medium, whereas *M. aurum* SCSIO 85700 maintained relatively robust growth under the same conditions.

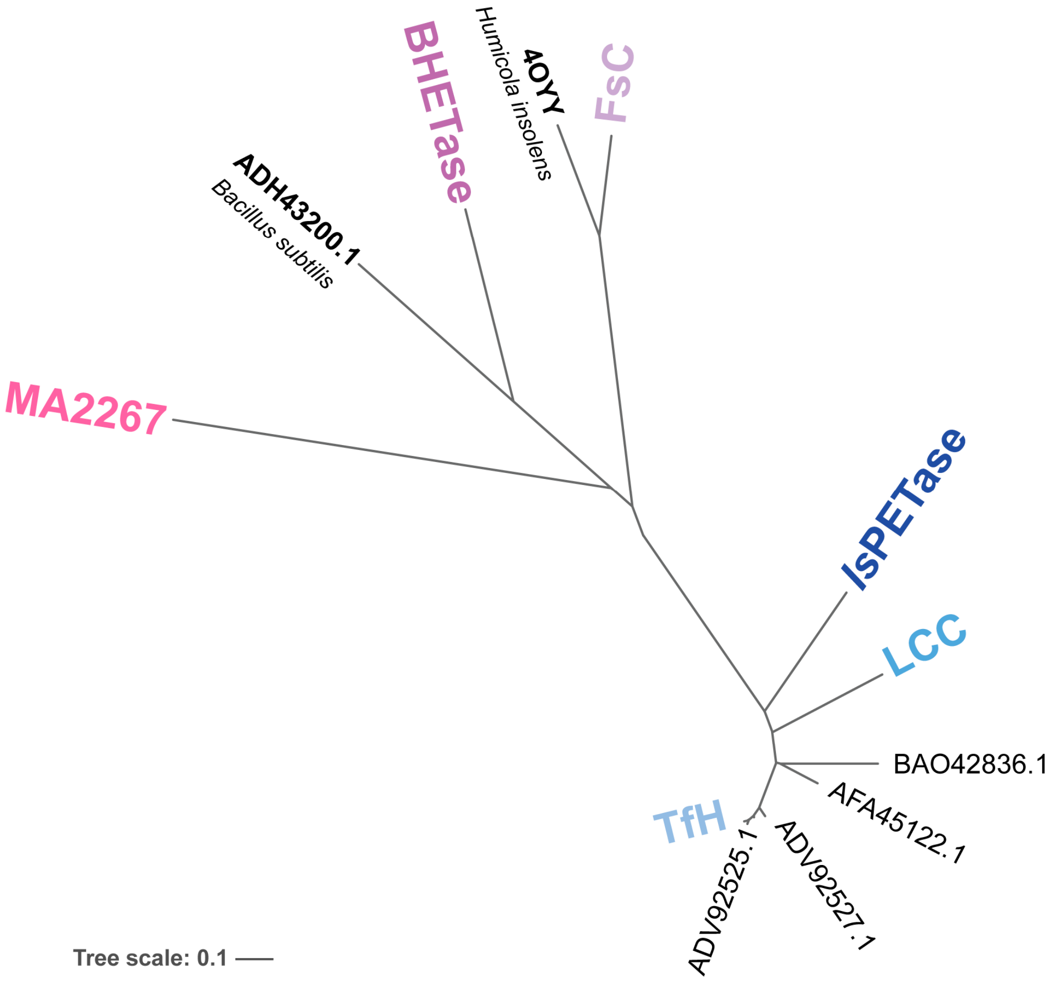

Figure S2. Phylogenetic analysis of the putative PET hydrolase MA2267.
